# Supplementary material for: The impact of the internationalization of China’s new retail industry on corporate performance—A moderating effect based on proprietary assets
Source: PLoS One. 2022 May 27;17(5):e0267825. doi: 10.1371/journal.pone.0267825 (PMC9140271; doi:10.1371/journal.pone.0267825)
Supplement: Attachment — Submitted filename: Response_to_Reviewer__20220403.docx [file pone.0267825.s002.docx]

PONE-D-21-28687

The impact of internationalization of China's new retail industry on corporate performance - a moderating effect based on proprietary assets

PLOS ONE

**Response to Reviewer**

1. Is the manuscript technically sound, and do the data support the conclusions?

The manuscript must describe a technically sound piece of scientific research with data that supports the conclusions. Experiments must have been conducted rigorously, with appropriate controls, replication, and sample sizes. The conclusions must be drawn appropriately based on the data presented.

Reviewer #1: Yes

Reviewer #2: Partly

Authors’ response:

Thanks for your suggestion.

1. We have strengthened the literature sources for 2016-2021 in the literature.

2. Our research design is rigorous, including strict design in content, structure, and method, and has certain quality standards in publishing SSCI.

2. Has the statistical analysis been performed appropriately and rigorously?

Reviewer #1: Yes

Reviewer #2: Yes

Authors’ response:

Thanks for your suggestion.

Our research data and structure have a certain quality, and it is acceptable to publish in SSCI.

3. Have the authors made all data underlying the findings in their manuscript fully available?

The [PLOS Data policy](http://www.plosone.org/static/policies.action#sharing) requires authors to make all data underlying the findings described in their manuscript fully available without restriction, with rare exception (please refer to the Data Availability Statement in the manuscript PDF file). The data should be provided as part of the manuscript or its supporting information, or deposited to a public repository. For example, in addition to summary statistics, the data points behind means, medians and variance measures should be available. If there are restrictions on publicly sharing data—e.g. participant privacy or use of data from a third party—those must be specified.

Reviewer #1: Yes

Reviewer #2: No

Authors’ response:

Thanks for your suggestion.

1.Our data source comes from the TEJ database in Taiwan.

2.TEJ(Taiwan Economic Journal co.,Ltd)

4. Is the manuscript presented in an intelligible fashion and written in standard English?

PLOS ONE does not copyedit accepted manuscripts, so the language in submitted articles must be clear, correct, and unambiguous. Any typographical or grammatical errors should be corrected at revision, so please note any specific errors here.

Reviewer #1: Yes

Reviewer #2: No

Authors’ response:

Thanks for your suggestion.

We let professional English teacher Jake to participate and help.

[www.academiccommunications.com](http://www.academiccommunications.com/).

The English is in line with the sentences and English standards published in SSCI.

5. Review Comments to the Author

Please use the space provided to explain your answers to the questions above. You may also include additional comments for the author, including concerns about dual publication, research ethics, or publication ethics. (Please upload your review as an attachment if it exceeds 20,000 characters)

Reviewer #1: I have enjoyed reading this paper. Introduction and literature review is well written. Methodology section is well designed. The authors had explained all the results in a critical fashion. This paper is suitable for publication.

Reviewer #2: The topic of the paper would be really interesting, however, there are some weaknesses.
Th abstract should be more clear and compact.
Introduction isn't complete enough,research questions and he context should be highlighted better.
Literature review is too short and only very few sources were processed in the paper.
The datasets are not provided, so I can not evaluate the validity of the results.

Authors’ response:

Thanks for your suggestion.

The parts we corrected are indicated by the red part.

1.We have further reviewed a number of papers (2016-2020) that are of relevance to the performance to re-write literature review in new retail. We have briefly described the shortcomings of extant literature and added an additional table (Table 1. Illustrative research summarizing the antecedents to performance in new retail) in the second paragraph of the “Introduction” section. Additional sentences and revisions are typed in red below.

2.(1) Abstract

**Purpose –** The purpose of this study is to examine the factors influencing China’s new retail industry on corporate performance. We mainly design the adjustment effect of the internationalization of its new retail industry on corporate performance and proprietary assets.

**Design/methodology/approach –** The framework is based on dynamic panel data of 590 listed companies in China’s new retail industry from 2007 to 2019.

**Findings –** We apply the concept of big data for the analysis and investigation, including the DOI explanatory variable for the degree of internationalization, Tobin's Q explained variable, and adjusted variable of proprietary assets (R&D intensity RDI, marketing intensity MI, and capital intensity CI). We found that the degree of internationalization of the new retail industry has a positive impact on corporate performance.

**Research limitations/implications –** The resultant findings only reflect the setting of China’s new retail marketplace. With the research model developed here in, future research can target cross-country studies of various forms of online and offline market sites to determine regional differences in the development of new retail activities.

**Originality/value –** The results show that the major new retail industry is a multi-channel concept that affects overall corporate performance. The authors focus on corporate performance, which is a moderating effect based on proprietary assets.

**Keywords:** new retail internationalization; corporate performance; proprietary assets

(2)Introduction:

The new retail industry is important to internationalization, and China's new retail model can serve as a reference for other countries, making it an interesting topic for research. We thus combined the concepts of marketing, financial management, and econometric methods to break through the traditional concept of only pure economics.

Lin et al. (2022) referred to Intelligent Capital, Organizational Learning, and Corporate Performance Influence Relationship. We know that the new retail industry uses the sharing of knowledge or information, but such a series of intangible asset processes are often overlooked as part of corporate performance.

In Table 1 we directly see the basic theories and findings concerning the topic of corporate performance.

Table.1 Illustrative research summarizing the antecedents to corporate performance

|  | Illustrative research | | Context | | Theoretical basis | Antecedents to performance in corporate performance | Key findings or propositions | |
| --- | --- | --- | --- | --- | --- | --- | --- | --- |
|  | HENGCHEN DAI et al. (2018) | | Others’ expectations to  manage impressions | | self-expectations theory | Affect, Impression | The findings suggest that affect and External Expectations and Impression  Management impact performance. | |
|  | Ehsan Moradi et al. (2021) | | Between business model innovation and open innovation | | Decision Theory | Effective organizational innovation is the  key to make and keep a competitive advantage | The findings suggest that innovation suggests that companies and businesses should be more flexible and open to innovation processes. | |
|  | Elfadil A. Mohamed et al. (2020) | | Impact of corporate performance on stock price predictions | | Expectations theory | Intelligence, GAs, sentiment analysis, and deep learning | The findings suggest that used a fused model of an ANN with PCA for  forecasting stock prices on the Tehran Stock Exchange. | |
|  | Fisher (1930) | | Long-run dynamic adjustments of the term structure  of interest rates | | expectation theory | Interest rates, dynamic adjustment | The results show that s investors’ expectations  about future spot interest rates affect current long-term interest rates. | |
|  | Liping Qian et al. (2018) | | Between online sellers and buyers | | Social capital theory | Trust | The results show that Hindering or enabling structural social capital to enhance buyer performance? | |
|  | Heinz-Theo Wagner et al. (2014) | | Between online sellers and buyers Value Co-Creation | | Social capital theory | IT alignment and  business value | The findings suggest that it drives operational alignment and IT business value. | |
| SHINTARO OKAZAK et al. (2017) | | Between social media knowledge sharing | | Social capital theory | | Knowledge Sharing | The findings suggest that Knowledge Sharing Social Media |  |

# Theoretical Analysis and Research Hypothesis

***2.1 Corporate performance***

Operating performance refers to a company's operating efficiency and operations performance during a certain time period. A company's operating efficiency level is mainly reflected in its profitability, level of asset operations, solvency, and follow-up development capabilities. Wei-Kang Wanga (2021) noted the Management characteristics and corporate performance of Chinese chemical companies. Yasas L. Pathiranage (2020) offered a literature review on organizational culture towards corporate performance.

*2.2 Degree of internationalization*

Hitt et al. (2006) pointed out that internationalization refers to the various operational activities adopted by manufacturers in expanding the sales of their products or services to transnational markets and transnational regions - that is, sales, R&D, manufacturing, and other operational activities done outside the home country can be called internationalization. Ziyi Wei et al. (2019) presented Chinese service multinationals’ impact on the degree of internationalization and performance. Carlos González (2019) spoke of DOI by the extent of a firm’s top managers’ international experience and the amount and cultural zone dispersion of the firm’s transnational board interlocking. Leandro Rodrigo Canto Bonfim et al. (2018) examined managers' international cognition and how risks affect company performance.

*2.3 Proprietary assets*

Proprietary assets refer to the ratio of shareholders' equity (capital plus profits) to assets, also known as self-owned capital ratio and property right ratio. They correspond to the debt ratio, which refers to the ratio of liabilities to assets. Jin K. IHan et al. (2001) presented capital requirements, cost advantages, switching costs, distribution access, and proprietary assets. In this paper, we investigate and analyze proprietary assets (R&D intensity (RDI), marketing intensity (MI), and capital intensity (CI)). Our primary objective is to determine whether the RDI, MI, and CI of a proprietary asset will influence the relationship between cause and effect variables in the overall architectural design.

# *5.1 Theoretical implications*

With the development of this novel research model, the theoretical contributions of this study to the literature are described as follows. We focus on the concept of

the impact of new retail management on company performance management by conducting a literature review, analysis, and possible integration within this theoretical framework. Specifically, although the degree of internationalization and proprietary assets are central in the literature, notable gaps remain in understanding their effect on corporate performance and their impact on decision-making by managers and executives.

We have further developed the social capital theory, decision theory, expectations theory, and self-expectations theory, and the contribution of this paper to them is as follows. Our research provides a clearer understanding of the impact of internationalization of the new retail industry on corporate performance. The social capital theory mainly talks about the whole process of operating online and offline transactions. There is no real intangible asset value except for tangible real value. In terms of marketing intensity, such a measure is often overlooked financially and economically, resulting in a lot of intangibles in marketing intensity that can help create a brand.

(3)Datasets: TEJ(Taiwan Economic Journal Co.,Ltd)

6. PLOS authors have the option to publish the peer review history of their article ([what does this mean?](https://journals.plos.org/plosone/s/editorial-and-peer-review-process#loc-peer-review-history)). If published, this will include your full peer review and any attached files.


If you choose “no”, your identity will remain anonymous but your review may still be made public.


**Do you want your identity to be public for this peer review?** For information about this choice, including consent withdrawal, please see our [Privacy Policy](https://www.plos.org/privacy-policy).

Reviewer #1: No

Reviewer #2: No

Authors’ response:

Thanks for your suggestion.

We do not require reviewers to disclose information.
